# Fundamentals for a Systematic Approach to Mild and Moderate Inherited Bleeding Disorders: An EHA Consensus Report

**DOI:** 10.1097/HS9.0000000000000286

**Published:** 2019-09-17

**Authors:** Francesco Rodeghiero, Ingrid Pabinger, Margaret Ragni, Rezan Abdul-Kadir, Erik Berntorp, Victor Blanchette, Imre Bodó, Alessandro Casini, Paolo Gresele, Riitta Lassila, Frank Leebeek, David Lillicrap, Diego Mezzano, Patrizia Noris, Alok Srivastava, Alberto Tosetto, Jerzy Windyga, Barbara Zieger, Mike Makris, Nigel Key

**Affiliations:** 1 Hematology Project Foundation, Vicenza, Italy, affiliated to the Hematology Department, San Bortolo Hospital, Vicenza, Italy; 2 Clinical Division of Hematology and Hemostaseology, Department of Medicine I, Medical University of Vienna, Austria; 3 Department of Medicine - Division Hematology/Oncology, University of Pittsburgh Medical Center Hemophilia Center of Western PA, Pittsburgh, USA; 4 Department of Obstetrics and Gynecology and Katharine Dormandy Hemophilia and Thrombophilia Centre, The Royal Free Foundation Hospital and University College London, London, UK; 5 Department of Translational Medicine & Centre for Thrombosis and Hemostasis, Lund University, Malmö, Sweden; 6 Department of Pediatrics, University of Toronto and Division of Hematology/Oncology, Hospital for Sick Children, Toronto, Canada; 7 3rd Dept of Internal Medicine, Semmelweis University, Budapest, Hungary; 8 Division of Angiology and Hemostasis, Faculty of Medicine, University Hospitals of Geneva, Switzerland; 9 Department of Medicine - Section of Internal and Cardiovascular Medicine jUniversity of Perugia, Perugia, Italy; 10 Coagulation Disorders Unit, Department of Haematology, Research Program in Oncology, Helsinki University Hospital;, Helsinki, Finland; 11 Department of Haematology, Erasmus University Medical Center, Rotterdam, the Netherlands; 12 Department of Pathology and Molecular Medicine, Queen's University, Kingston, Canada; 13 Department of Hematology-Oncology, School of Medicine, Pontificia Universidad Católica de Chile; 14 Department of Internal Medicine and Medical Therapy, University of Pavia and Department of Medical Sciences and Infectious Diseases, IRCCS Policlinico San Matteo Foundation, Pavia, Italy; 15 Department of Haematology, Christian Medical College, Vellore, India; 16 Hemophilia and Thrombosis Center, Hematology Department, S. Bortolo Hospital, Vicenza, Italy; 17 Department of Hemostasis Disorders and Internal Medicine, Institute of Hematology and Transfusion Medicine, Warsaw, Poland; 18 Department of Pediatrics and Adolescent Medicine, Division of Pediatric Hematology and Oncology, Medical Center – University of Freiburg, Faculty of Medicine, Germany; 19 Sheffield Hemophilia and Thrombosis Centre, Royal Hallamshire Hospital, Sheffield, UK; 20 Department of Medicine, Division of Heamtology-Oncology, University of North Carolina at Chapel Hill, NC, USA.

## Abstract

Healthy subjects frequently report minor bleedings that are frequently ‘background noise’ of normality rather than a true disorder. Nevertheless, unexpected or unusual bleeding may be alarming. Thus, the distinction between normal and pathologic bleeding is critical. Understanding the underlying pathologic mechanism in patients with an excessive bleeding is essential for their counseling and treatment. Most of these patients with significant bleeding will result affected by non-severe inherited bleeding disorders (BD), collectively denominated mild or moderate BD for their relatively benign course. Unfortunately, practical recommendations for the management of these disorders are still lacking due to the current state of fragmented knowledge of pathophysiology and lack of a systematic diagnostic approach. To address this gap, an International Working Group (IWG) was established by the European Hematology Association (EHA) to develop consensus-based guidelines on these disorders. The IWG agreed that grouping these disorders by their clinical phenotype under the single category of mild-to-moderate bleeding disorders (MBD) reflects current clinical practice and will facilitate a systematic diagnostic approach. Based on standardized and harmonized definitions a conceptual unified framework is proposed to distinguish normal subjects from affected patients. The IWG proposes a provisional comprehensive patient-centered initial diagnostic approach that will result in classification of MBD into distinct clinical-pathological entities under the overarching principle of clinical utility for the individual patient. While we will present here a general overview of the global management of patients with MBD, this conceptual framework will be adopted and validated in the evidence-based, disease-specific guidelines under development by the IWG.

## Introduction

The hemostatic system maintains blood fluidity while reducing the risk of bleeding or thrombosis through a finely tuned and integrated interplay of an intact vasculature and its endothelium with platelets, blood coagulation and fibrinolysis. In principle, any genetic abnormality in any component - the function of which could not be fully replaced by the redundancy of the system - will appear phenotypically as a tendency towards excessive bleeding or a thrombotic risk. Minor bleeding manifestations, although often alarming, are common in otherwise healthy people who can sustain major hemostatic challenges without danger.^1,2^ However, they may be also indicative of several pathophysiologically-defined bleeding disorders (BDs) characterized by a mild or moderate bleeding tendency. During the past decades, as a result of progress in laboratory and genetic diagnosis, an increasing number of such BDs has been described.

Inherited BDs may be due to quantitative or qualitative defects of one or more of the components of the hemostatic system or vessel wall collagen and other matrix components, and have been referred to as mild or mild/moderate bleeding disorders, using the acronym MBD.^3–8^ Currently, the screening and diagnosis of inherited MBDs remain challenging and the assessment of the magnitude of future bleeding risk and of the burden of these disorders on quality of life (QoL) have been poorly investigated, apart from the few studies in adults with VWD^9^ and in women with heavy menstrual bleeding as their dominant symptom.^10^ Hence, screening and diagnostic work up of patients for a suspected MBD tend to be based on local practices rather than guided by sound clinical, laboratory or genetic criteria.

To address this gap, the European Hematology Association (EHA) established an International Working Group (IWG) to develop a series of guidelines on MBDs. The IWG agreed to maintain this acronym, where M stands for “mild to moderate” to encompass the wide variation of bleeding phenotypes to indicate an overall milder bleeding phenotype when contrasted to the more rare severe hemostatic disorders that, if untreated, may cause life- or organ-threatening hemorrhages.

The aim of this first communication is to define the basis of a systematic, uniform approach to MBDs and to provide a consensus on the principles underlying the following guidelines that will be specifically dedicated to: non-type 3 von Willebrand Disease; Coagulation and fibrinolytic system disorders; Platelet and vascular disorders; Bleeding of unknown cause.

The IWG agreed that these guidelines should assure clinical utility in terms of a real benefit for the patient and/or family members while simultaneously helping physicians to exclude false positive or useless diagnoses, thereby avoiding the risk that healthy subjects become disadvantaged by the stigma of a hereditary disorder while providing guidance for the management of those confirmed to have MBDs.

At the same time, the IWG is well aware that current diagnostic technologies suffer from major limitations and that the genetic approach is increasingly used but still in development, so that areas of future research should be identified.

## Methodology

The IWG included 20 members selected for their recognized expertise in research and clinical practice, with consideration of a wide geographic representation to provide an international perspective. EHA verified the absence of relevant competing conflict of interests of the IWG and supervised the project with regard to compliance to, and consistency with, the methodology adopted by its Committee on Guidelines. EHA provided organizational and logistic assistance and funding for face-to-face meetings or teleconferences.

The IWG agreed to hereby present a self-standing introductory paper on the various methodological aspects for the definition and classification of inherited MBDs, and for a standardized approach to diagnosis and management. With this background, a series of disorder-specific evidence-based guidelines will be produced using a Delphi-like approach when evidence-based recommendations could not be derived from a systematic literature review. For this preliminary document, it was agreed that a systematic review of literature was not necessary. However, to share a common domain of knowledge, the IWG coordinator (FR) made available an initial list of references dealing with MBDs or with the general principles that should guide a diagnostic and management process. A provisional list of potential candidate MBDs was also proposed to represent a concrete domain for discussion and analysis and to facilitate the planning of future disease-specific guidelines and related systematic literature review (Supplemental Table 1, Supplemental Digital Content). As explained in the footnote to Supplemental Table 1, this provisional list should in no way be intended as a list of established MBDs. After agreement on the main scope and structure of the document, a consensus was progressively reached through an iterative process, guided in a no-directive way by the coordinator of the panel. This process required at least 10 rounds of progressive drafts circulated among all IWG members highlighting all amendments and suggestions. In addition to electronic exchanges, three face-to-face meetings were held in conjunction with EHA or ASH meetings from 2016 to 2018 and a final conference call was made. Finally, the submitted version was unanimously approved by all authors.

## Definition of MBDs: clinico-pathological criteria and proposal for standardization

A bleeding phenotype sufficiently distinctive to be considered as a disease status, but of much less severity in comparison to severe bleeding disorders, was taken as the major unifying factor of MBDs. Representative examples are patients with type 1 VWD; mild or moderate hemophilia or hemophilia carriers; heterozygous deficiencies of specific clotting factors; deficiency of fibrinolysis inhibitors or some quantitative and/or qualitative platelet disorders. In contrast, a distinctly severe phenotype was considered to be associated with severe hemophilia, type 3 VWD, some homozygous recessive coagulation defects or some platelet function defects such as biallelic Glanzmann Thrombasthenia (GT) and Bernard-Soulier syndrome. However, apart from these and a few other examples, the IWG acknowledged the lack of a precise definition delimiting the MBD category. Indeed, we still do not have consensus on the explicit clinical criteria for making decisions, due to the continuous spectrum of increasing severity from normal subjects to those with mild/moderate and severe disorders. The IWG agreed that combining these disorders under the single, although imperfect, category of MBDs, together with a standardization of definitions and terminology will facilitate ongoing efforts to streamline a systematic approach to these disorders and to develop comprehensive and reliable diagnostic algorithms that result in optimal management strategies.

Notably, the grouping of MBDs is based more on bleeding phenotype than on laboratory or genetic features, although it is acknowledged that this grouping does not point to a specific diagnosis, nor does it generally predict the risk of future bleeding. Indeed, only distinct mechanistic characteristics, as revealed by laboratory or genetic investigations, confer disease specificity to the different MBDs, thus substantially contributing more appropriately to management and counseling. Accordingly, the IWG adopted clinico-pathologic criteria to classify MBDs as distinct disease entities, qualified by a unique set of hemostatic and/or genetic abnormalities (Table 1).

**Table 1:**
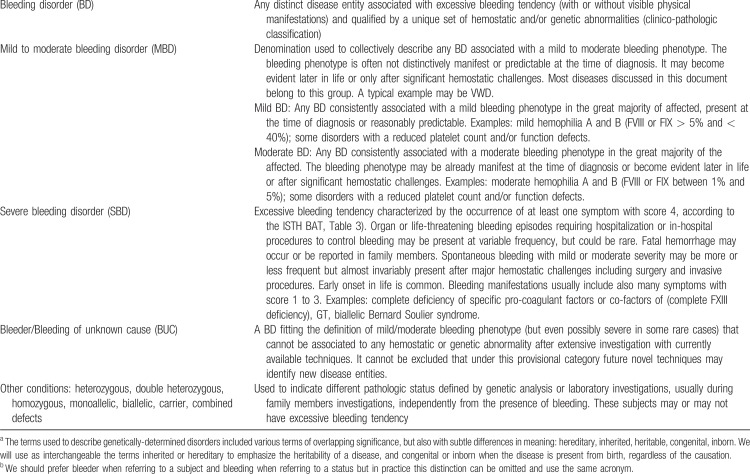
Definition of Inherited/Congenital Bleeding Disorders (BD), Bleeders of Unknown Cause (BUC) and Other Conditions^a^^,^^b^

However, we acknowledge that a sizable proportion of patients without any demonstrable underlying hemostatic or genetic abnormalities may have a bleeding phenotype as severe as patients with an established MBD, in whom a specific laboratory or genetic diagnosis is apparent. These subjects are commonly classified as BUC (bleeders/bleeding of unknown cause).^11^

### Clinical aspects: definition of bleeding phenotype

The IWG agreed to use the standardized definitions of 14 distinct symptoms and their severity scale (from 1 to 4) adopted in the Bleeding Assessment Tool (BAT) endorsed by the International Society on Thrombosis and Haemostasis (ISTH).^12^

#### Bleeding symptoms definition and severity-based classification

The ISTH-BAT makes a clear distinction between “trivial” bleeding, that should not be considered, and “true” bleeding manifestations, which may be representative of a disease and that, according to their severity, are scored in the range of 1 to 4. Minimal criteria for defining a bleeding symptom as significant (non-trivial) and the relative score according to the ISTH-BAT scale are shown in Tables 2 and 3 (adapted from Rodeghiero et al^12^). The recommended definitions and terminology for describing the severity of bleeding symptoms and of bleeding phenotypes are shown in Table 4. Since the frequency of bleeding is not accounted for as a primary grading factor by the ISTH-BAT, we have introduced a new category of nuisance bleeding to account for patients suffering from frequent and disturbing bleeding symptoms that, individually, could be considered trivial. This category has been borrowed from definitions proposed for patients treated with antithrombotic therapy^13,14^ to describe bleeds that impact patient QoL for their frequency and recurrence (Table 4).^15^

**Table 2:**
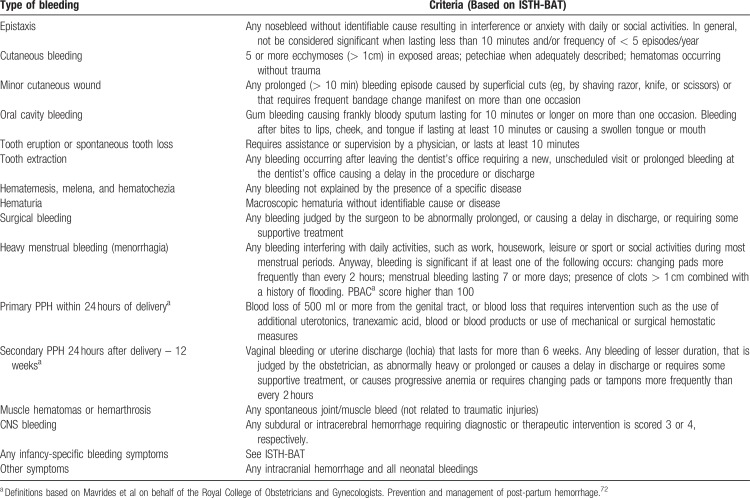
Minimal Criteria Defining a Bleeding Symptom as Significant (non-trivial)

**Table 3:**
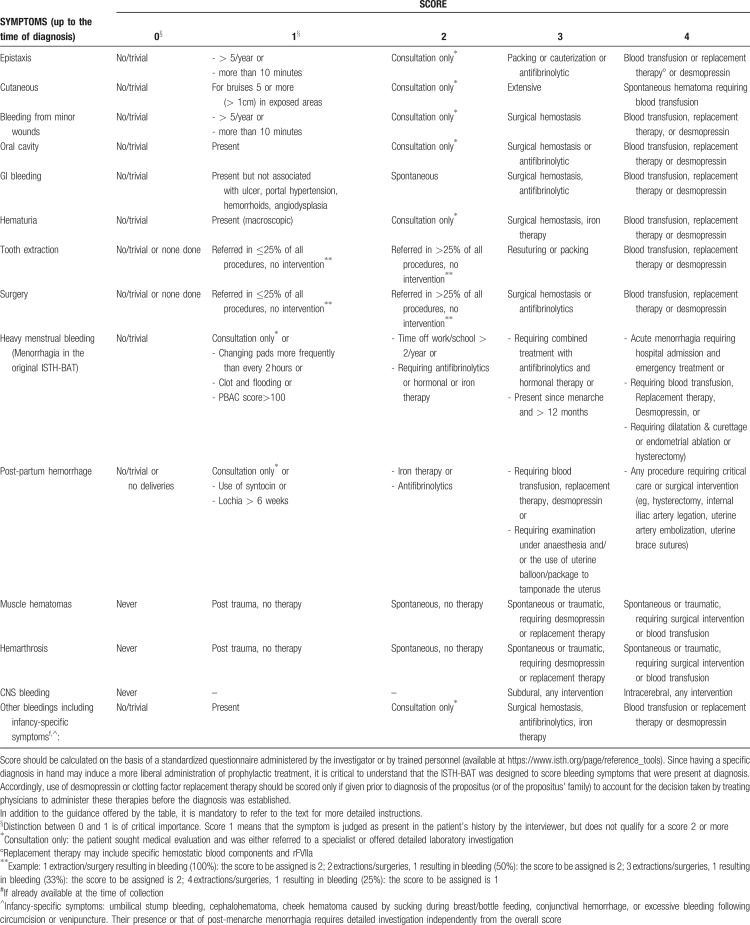
Severity Score Assigned to Distinct Bleeding Symptoms According to the ISTH-BAT Scale, Only Episodes before Diagnosis should be Considered

**Table 4:**
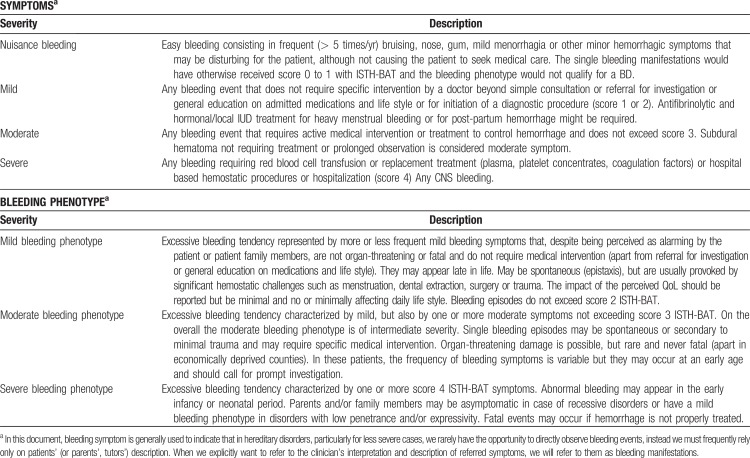
Recommended Definitions and Terminology for the Description of the Severity of Bleeding Symptoms and Associated Bleeding Phenotypes

While some modifications in grading the bleeding symptoms have been proposed to improve the utility of the ISTH-BAT,^16^ the IWG members agreed that they do not have any relevant impact on the description and classification of bleeding symptom severity, provided that only episodes before diagnosis are considered.

With this caveat, the ISTH-BAT can also be used for the investigation of family members, recognizing that the bleeding phenotype can be highly variable even in family members carrying the same genetic defect. It would be desirable to extend the investigation beyond first-degree relatives, but we acknowledge that this is often not possible and raises questions about privacy. The propositus, or parents, may be encouraged to approach other family members beyond first-degree.

Notably, MBDs do not protect against the occurrence of thrombotic events in specific clinical settings, as will be discussed in specific guidelines.

#### Physical examination and exclusion of acquired conditions

Physical examination may be relevant for the detection of particular signs such as petechiae on the skin or visible mucosal bleeding, large ecchymoses in exposed areas (pointing to senile purpura), hemarthrosis, joint hyperflexibility, facial or skeletal anomalies or telangiectasia. To diagnose vascular disorders, the Beighton score may be used to assess hypermobility.^17^ The distribution and type of bleeding manifestations may raise the suspicion of non–accidental injury (eg, child or adult battered syndrome) or of psychogenic or self-inflicted (factitious) lesions (eg, Munchausen syndrome), or bilateral periorbital purpura (“raccoon eyes”) in AL amyloidosis. In addition, physical examination and a comprehensive general clinical history could help to exclude that bleeding is associated to acquired conditions such as uremia, liver disease, Cushing syndrome, hypothyroidism, hypertension and anemia, to name a few.

### Laboratory and genetic aspects

#### Laboratory aspects

**FIGURE 1:**
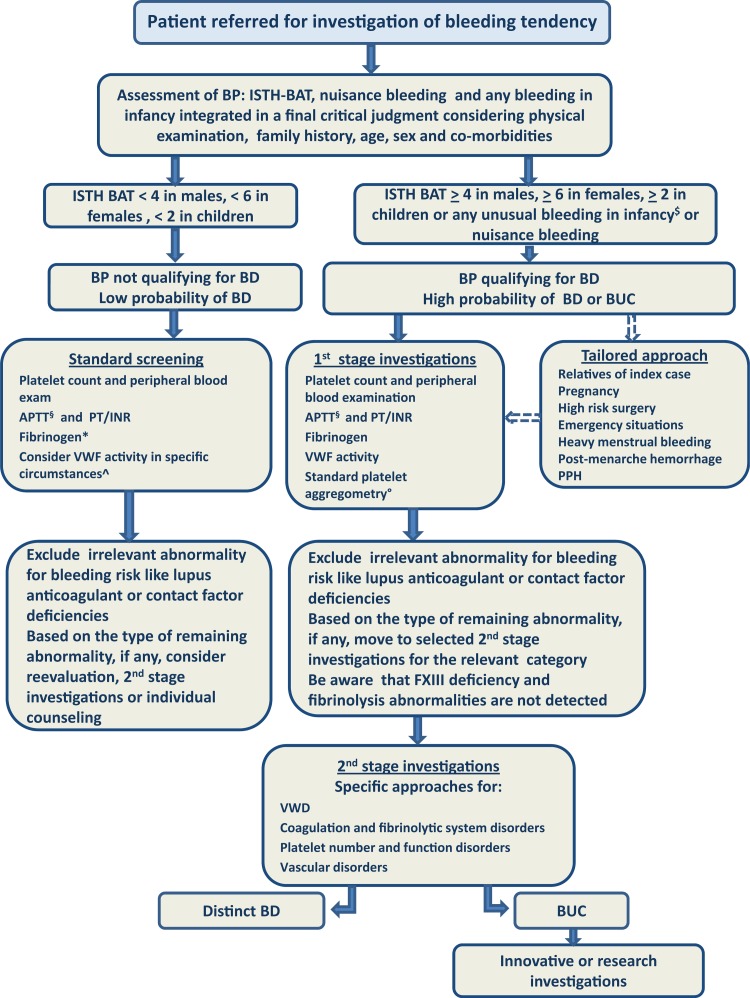
**A proposed diagnostic approach for patients referred for investigation of bleeding symptoms**. Standard screening is performed in all referred subjects to confirm negative results of previous tests or to clarify any abnormalities detected. Patients with a qualifying bleeding phenotype should undergo 1st stage investigations and, on the basis of the results, be directed to 2nd stage investigations, distinct for VWD, other coagulation and fibrinolytic disorders and platelet number/function disorders. Vascular disorders should be considered based on physical examination. Some tests could be anticipated and a full investigation postponed under particular circumstances requiring a tailored approach. Patients with a doubtful history should undergo standard screening including, in particular circumstances, measurement of VWF activity. A further stage including innovative and/or research investigations can be considered for BUC. All BD are considered together in the diagnostic process, as it is intended to be appropriate as a general approach to patients at risk of bleeding. BP, bleeding phenotype; BD, bleeding disorder; BUC, bleeding of unknown cause. Broken arrows indicate an alternative approach tailored for specific circumstances, as discussed in the text. ^$^Includes: umbilical stump bleeding, cephalohematoma, cheek hematoma caused by sucking during breast/bottle feeding, conjunctival hemorrhage, or excessive bleeding following circumcision or venipuncture and post-menarche menorrhagia. ^§^If not sure that APTT is sensitive to FVIII and FIX < 40-50%, specific assays for FVIII and FIX are required. ^∗^Thrombin time can be used if fibrinogen activity assay is not available or is not being used in the clinical laboratory. ^^^Low VWF level may be a risk factor for bleeding in subjects not meeting the criteria for a bleeding phenotype qualifying for a MBD. In specific circumstances, in case of doubtful bleeding history in young people undergoing surgery (e.g., tonsillectomy) or other hemostatic challenges, the presence of low VWF may guide specific replacement therapy. Standard platelet aggregometry (LTA) is imperfect and technically challenging but remains the best screen available for a mild/moderate inherited platelet disorder. Quality assurance testing and use of a standardized battery of agonists are critical. Where available, lumi-aggregometry may be preferred because rare platelet disorders may have normal platelet aggregation, but abnormal granule release, like the Hermansky Pudlak syndrome; however the disease can be suspected by physical examination.

A detailed investigation of the laboratory phenotype beyond the results of first-stage investigation (Fig. 1) may indicate the underlying pathogenic mechanism and, depending on the nature of the disorder, guide treatment and, in some cases, be sufficiently predictive of the patient's future bleeding risk. FVIII or FIX deficiencies may be the most representative examples since a good correlation between the residual clotting activity and the bleeding phenotype seems well established.^18^ So, the classical laboratory-based distinction between severe, mild and moderate hemophilia is still maintained in the literature and will be here adopted (Table 1), even if it has been recently questioned, with a proposal to move from a laboratory-based to a bleeding phenotype-based classification,^19^ or to a much more complex laboratory and genetic investigation.^20^ Similarly, it remains questionable if patients with a marginally low VWF level (between 30% and 50% of normal range) may have a significant bleeding phenotype as reflected by the ISTH-BAT score.^21^ In most MBDs laboratory abnormalities might prove inconsistent in predicting the risk of bleeding. The IWG agrees that in the absence of a qualifying bleeding phenotype, it is inappropriate to consider subjects as being affected by a MBD unless the qualifying laboratory assay has a very high likelihood for a distinct disorder.

#### Genetic aspects

Even genetic abnormalities, now detectable on a massive scale, cannot be invariably linked to a precise clinical and laboratory phenotype. Cutting-edge technologies in genetics and genomics may contribute to group disorders previously classified as distinct entities into the same category or identify new disorders among those previously considered the same entity. However, it remains unproven if the non-targeted application of these technologies will be of clinical utility in daily practice.

The phenotypic effects of genetic abnormalities may be partially compensated or masked by the individual genetic backgrounds. The clinical phenotype of most bleeding disorders is rarely the result of a monogenic defect, but it is often influenced by the interaction between different genes/polymorphisms and/or between genetic and acquired factors. This complex genetic interplay may aggravate or alleviate the severity of the bleeding phenotype in general population.^22^ Interesting examples in bleeding disorders may be found in variations in genes that affect VWF levels including CLEC4 M or STXBP5^23,24^ or in the presence of prothrombotic mutations, such as factor V Leiden that may attenuate the phenotype of bleeding disorders such as in hemophilia.^25^ Furthermore, the impact of a single mutation may be even less predictable, as in the case of multi-subunit molecules such as fibrinogen or multimeric proteins such as von Willebrand factor (VWF). In these cases, depending on the particular genetic defect, the variable inclusion of the abnormal subunit or monomer into the final product might produce a continuous spectrum of residual activity from zero to the lower limit of normal, so that in these instances the contribution of genetic studies appears of limited practical relevance [eg, VWD]. Even more complex is the diagnostic role of genetics in vascular disorders or hereditary thrombocytopenias, with or without additional functional defects and/or syndromic features. In some of these patients, the incidental discovery of genetic mutations may be associated with an increased future risk of hematological malignancy.^26^

### Bleeders of unknown cause

In a sizable proportion of patients with a qualifying bleeding phenotype - and sometimes with a family history of bleeding - no hemostatic or genetic abnormality can be found, even after an extensive investigation. These cases have been variably referred to as “bleeding/bleeders of unknown cause (BUC)”.^4,11^ While currently BUC is meant to describe a true BD despite the absence of distinct laboratory findings, with the improvement of molecular characterization or of other laboratory technologies, some previous BUC could receive a clinico-pathological diagnosis. For example, two such new autosomal dominant disorders have been recently identified in large kindred with techniques or measurements available only in dedicated research laboratories:^27^ the first caused by an abnormal short form of Factor V associated with severe bleeding manifestations, in which APTT and PT were prolonged but the levels of all known coagulation factors were normal (East Texas bleeding disorder);^28,29^ the second, associated with normal screening assays (but reduced prothrombin consumption) caused by a novel mutation in the thrombomodulin gene, which seems to fit our definition of MBD.^30^

## Clinical utility should guide patients’ investigation

Diagnostic tests and procedures are justified only if a net health benefit for the patient is expected from such testing. Tests should fit the purpose and the context in which they are used.^31^ For example, patients may be referred for specific circumstances such as urgent preoperative screening; emergency circumstances in the obstetric, surgical or trauma setting; incidental laboratory findings or as relatives of a subject with a known hemostatic disorder. From the global healthcare perspective, the concept of clinical utility is embedded in the broader concept of value, defined as outcomes for the patient relative to the cost required to produce them. This efficiency criterion - increasingly adopted by healthcare delivery organizations - is endorsed by the IWG once utility for patients remains the overarching governing goal.^32^ Accordingly, we will give major emphasis on patient short- and long-term outcomes rather than costs, but comments on avoiding redundant, poorly informative or useless testing will also be provided to prevent futile costs. Clinical utility implies analytical validity (in terms of precision or reproducibility and accuracy) and clinical validity (in terms of sensitivity, specificity, positive [PPV] and negative predictive values [NPV]) as applied to the various populations under study. The level of some hemostatic factors may be influenced by physiologic conditions. For example, VWF and FVIII activity increases with age,^33,34^ but without leading to a mitigation of symptoms.^35^ In women, VWF levels vary during the phase of menstrual cycle^36^ and increase in association with hormonal therapy and during pregnancy (particularly in VWD type 1).^37,38^ In these instances, the lowest historical VWF is assumed for diagnosis, but the actual level should be considered for individualizing the replacement treatment. Moreover, normal ranges in the first year of life are different from adults, and in toddlers may be influenced by recent infections or by the transient occurrence of a weak lupus anticoagulant or acquired FXII deficiency that may disappear over the years, resulting in undue alarming prolongation of the APTT. Finally, subjects with blood group O have lower VWF levels, but it remains unknown if this has a clinical effect on patients with established VWD. In summary, all these factors influence the clinical utility of tests and deserve a critical consideration.

Currently, an extensive search for underlying genetic mutations is possible using high throughput sequencing facilitated by commercial or institutional platforms with a growing list of genes,^39^ but the IWG does not support a blind genetic search for putative mutations outside the research setting. The IWG noted that presently very few genetic tests meet clinical utility criteria. In particular, for some MBD, like inherited platelet disorders, a combination of clinical and laboratory criteria may be sufficient for a conclusive diagnosis. For others, genotyping is not mandatory but may be advisable, for instance because a genotype/phenotype correlation has been established. For a few cases, when the clinical and laboratory picture is disorienting, functional alterations are heterogeneous, or characterization is uncertain, because too few cases have been described (eg, GT variants, cPLA_2_ deficiency) genotyping may be recommended.^40^ Exceptions may be cases of particularly difficult diagnostic classification, where an exact diagnosis has practical implications such as in the differential diagnosis of mild hemophilia A vs VWD type 2N in males, hemophilia A carrier status vs. VWD type 2N in females, VWD type 2B vs platelet-type (pseudo) VWD^41^ or in some inherited thrombocytopenias. In the most frequent form of inherited thrombocytopenia, MYH9-related disease, specific MYH9 variants can predict the likelihood of severe bleeding, renal disease and deafness.^42^ In some other instances, molecular diagnosis may help to identify thrombocytopenias responsive to a thrombopoietin receptor agonist (TPO-ra).^43^ However, genetic testing raises serious ethical, legal and social implications, since results may cause stigmatization, family discord, and psychological distress and should be performed with particular caution and with the highest standards of informed consent and privacy. Access to healthcare providers specifically trained to interpret results of genetic testing and to convey appropriate counseling should be offered. Particular attention to these issues should be paid when genetic testing and counseling apply to children.^44,45^ This is particularly true in some conditions like for example familial thrombocytopenias with ANKRD26, RUNX1, ETV6 mutations that may predispose to hematologic malignancies or herald evolution into bone marrow failure (MPL and MECOM mutations).^26^ In these instances, clinical utility of diagnosis remains contentious, given the fact that currently no treatment is available for these patients and as not all affected patients will develop leukemia, and might have a negative impact, particularly in children.^46^

Under these premises, the IWG agreed that a multi-stage standardized diagnostic approach could be a more useful and effective strategy to investigate most patients, reserving contextualized approaches for specific situations such as those mentioned previously.

## Proposal for a systematic diagnostic procedure

### General criteria for initiating multistage laboratory investigation

The underlying rationale for the proposed diagnostic strategy (Fig. 1) is dual: to have sufficient sensitivity (and NPV) to avoid missing patients at risk of future bleeding, who may benefit from specific treatments; at the same time, to have sufficient specificity (and PPV) to spare healthy subjects further clinical or laboratory testing.

Positive and negative outcomes for the individual patient of a specific diagnosis of MBD need consideration when initiating any extensive investigation (Table 5). The IWG agreed that it is useful to perform a standard screening (APTT, PT/INR and platelet count) before moving to first-stage investigation and further. These simple tests could either confirm previous results, or point out incidental findings not bearing a risk of bleeding, like pseudothrombocytopenia or defects in some contact phase clotting factors or the presence of a transient lupus anticoagulant. The multi-stage investigation should generally be reserved for patients with a pathologic bleeding phenotype or in whom standard screening reveals relevant abnormalities.

**Table 5:**
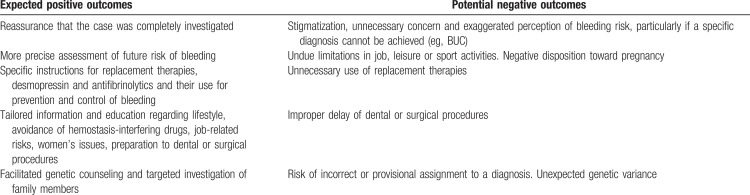
Positive and Negative Outcomes for the Patient after Extensive Investigations for the Specific Diagnosis of a MBD

Traditionally, pathologic bleeding phenotype is assessed by history-taking. However, in the absence of universally accepted criteria for a standardized bleeding history, the IWG proposed the use of the ISTH-BAT as the main reference standard to identify subjects deserving multistage investigation, based on a global bleeding score above a decisional threshold. To overcome some limitations, patients with nuisance bleeding, as defined in Table 4, were also included in the multistage investigations considering that they would not be captured by the ISTH-BAT, which scores the severity and not the frequency of bleeding. In addition, the IWG recognized that the definition of an abnormal bleeding phenotype lies ultimately in the subjective judgment of an expert clinician, integrating the many confounding factors, such as the age of the subject and age at first non-trivial bleeding, comorbidities such as hypertension, number of previous hemostatic challenges, recall biases, or the presence of other family members with bleeding. Acquired conditions should be excluded upfront. The discussion here is limited to standard screening and first-stage examination. Discussion on second-stage investigations will be deferred to the evidence-based disease-specific guidelines, which might lead to modifications/integrations of the multistage approach here proposed.

The clinical utility of global tests including thromboelastography on whole blood or platelet-rich plasma, measurements of thrombin potential/generation.^47,48^ platelet function analyzer (PFA)-100 and the skin bleeding time^49,50^ remains unestablished and these tests were not considered appropriate to direct further investigations.

#### Critical aspects in the use of ISTH-BAT in decision making

In the ISTH-BAT, a cumulative score is obtained by summing the worst ever recorded score for each symptom (Table 3). Thresholds can be set at variable sensitivity (rate of detection of patients with a bleeding disorder requiring investigation, true positive rate) and specificity (rate of healthy people who are correctly identified as not needing investigation, true negative rate). In this way, standardization and comparison of different studies is facilitated. The cumulative score will serve as a proxy representation of the overall severity of bleeding phenotype. It is reasonable to assume that the severity of the bleeding history and the accumulated outcomes with hemostatic challenges correlate with the bleeding risk. This is in keeping with the findings of two major studies but not invariably demonstrated. In a 1-year prospective study in 769 patients with various types of VWD, a bleeding score >10 was the only independent predictor of the need for future treatment with DDAVP or concentrates.^51^ Moreover, the ISTH BAT has been demonstrated to be highly predictive of postsurgical bleeding for patients with inherited platelet disorders, with an ISTH BAT ≥6 associated with strongly increased bleeding risk.^52^ On the other hand, it should be pointed out that in some studies no differences in bleeding score have been found between patients with and without an established MBD.^4,53^ Anyway, it appears that the cases missed by the ISTH-BAT would have been detected by the standard screening and first-stage examination, as described in Figure 1. In this regard, under the assumption that any test is justified by patients’ clinical utility, the aim of the BAT is not to demonstrate a strict correlation between any identifiable BD and bleeding score, but to include in the diagnostic process those individuals that could benefit from being identified as having a significant risk of future bleeding (sensitivity), while maintaining a high degree of specificity.

Other quantitative BATs have been proposed, with slightly different scales,^54^ or adapted to a pediatric population,^55^ or in a condensed form;^56^ all are derived from a model initially established in obligatory carriers of type 1 VWD, also known as the Vicenza Bleeding Questionnaire.^57^ Critical reviews have been published.^16,58^ In particular, a recent systematic review on the diagnostic accuracy of various BATs showed that specificity and sensitivity greatly varied, offering limited diagnostic value to the work up of patients referred for bleeding evaluation in clinical practice.^59^ However, this analysis cannot demonstrate if the cases not captured by the ISTH-BAT were of clinical importance or would not have been detected by standard screening, which we propose to be applied to all referred patients (Fig. 1). Notably, both the ISTH-BAT (symptoms scored from 1 to 4) and the MCMDM-1VWD BAT (symptoms scored from −1 to 4, used in wide European series of studies)^54^ yielded the same cumulative score values at the 2.5 percentile of the normal population (0–3 for males, 0–5 for females, 0–2 for children), as shown by an extensive study in 500 healthy individuals of different ages.^60^ Accordingly, this information can now be used to objectively assess bleeding symptoms as “normal” or “abnormal”. Therefore, the cut-off for a positive or abnormal bleeding score is ≥4 in adult males, ≥6 in adult females and ≥3 in children, and is associated with a specificity of 97.5%.^60^ The bleeding score was the same in males and females, once menorrhagia and post-partum bleeding were excluded. The IWG proposed the adoption of these decisional limits in future studies and in clinical practice as sufficient to qualify a bleeding phenotype as worthy of multistage investigation. In this way, subjective judgment is at least partially overcome. In a prospective study of patients referred for bleeding history to a tertiary center, using an abbreviated ISTH-BAT, a score ≥4 proved to have high specificity (0.81) and acceptable sensitivity (0.41) with NPV% of 99.3 and 84.5 in a clinical setting of low (1%) or moderate (20%) prevalence and was significantly and independently associated with the presence of an odds ratio of MBD of 6.6.^61^ In clinical practice, to increase the sensitivity of ISTH-BAT, we recommend that patients with any grade ≥1 bleeding at very young age or with a history of infancy-specific bleeding symptoms (as specified in the footnote to Table 3) are routinely investigated. These symptoms greatly contributed to the classification of the severity of bleeding phenotypes in a pediatric population with VWD^62^ and their occurrence in infancy requires detailed investigation independently from the overall score of the ISTH-BAT. Patients with nuisance bleeding, independent of the ISTH-BAT score, also deserve multistage laboratory investigation. However, this standardized approach might be fallacious, particularly in young people or in subjects with few hemostatic challenges in their history, so that a sound clinical judgment can never be omitted.^59^

#### Tailored approaches

Different contexts might require a tailored approach and a different hierarchy of laboratory tests. In investigating the relatives of an index case with a known disorder, often a single specific measurement or the detection of a specific mutation might be sufficient and the application of the ISTH-BAT would be adjunctive, serving primarily to more precisely define individual bleeding risk. In emergency situations, the hematologist should switch to an immediate diagnostic approach to exclude acquired BDs (eg, acquired hemophilia) or inherited disorders that could have been missed (eg, FXIII deficiency, VWD or platelet disorders). Examples are major bleeding after surgery or post-partum, disproportionate bleeding after a minor hemostatic challenge, profuse bleeding during pregnancy (once major obstetric causes of bleeding have been excluded). Finally, in some adults the bleeding history remains of dubious significance, as in patients already exposed to hemostatic challenges with inconsistent bleeding manifestations. In these cases, we endorse the time-honored practice suggested more than three decades ago by the late Rapaport,^63^ that normality of minimal screening including APTT, PT/INR (performed with a sensitive reagent capable of detecting factors concentration below 40–50%) and platelet count might be sufficient to exclude major hemorrhages. Limiting investigation to a few essential tests will generally exclude the most severe congenital or acquired BDs. However, for high-risk surgery, second-stage tests are advisable. Unselected coagulation testing with the goal of assessing bleeding risk prior to surgery in healthy subjects, although widely practiced, is discouraged on the basis of a systematic literature search.^64^

## General principles of management

### Counseling and patients’ education

It is of critical importance that treating physicians provide information on the various steps of investigation and on the final diagnosis to patients. The benefits of the diagnostic outcome should be maximized and the possible negative consequences openly addressed (Table 5). This goal can be achieved only with a complete and understandable communication. The concerns of the individual and/or family members should always be taken into consideration and addressed in a supportive and non-directive/objective manner. The relationship between bleeding history, laboratory or genetic testing and future risk of bleeding should be clarified and integrated and should constitute the basis for personalized counseling and education. Adults with a bleeding history of dubious significance and normal screening tests should be suggested to avoid a liberal use of drugs interfering with hemostasis and reassured that unexpected bleeds could be easily managed with tranexamic acid and/or local procedures. Of note, patients with MBD are not exempted from thrombosis so that antithrombotic agents may be required, making an assessment between risks and benefits very difficult even to experts, due to the complete lack of any evidence. Second-stage investigations are always recommended in case of high risk surgery.^63^ Depending on the specific diagnosis, information on available treatments including antifibrinolytic agents, desmopressin, platelet transfusion, TPO-ra, recombinant activated FVII, and replacement therapies should be provided. Detailed instructions should include measures for prevention or control of spontaneous bleedings such as epistaxis or gum bleeding and at-risk situations, like dental and surgical procedures or traumatic events. Issues specific for women (menarche, menstruation, pregnancy) may be of particular importance as discussed in the dedicated section. In case of bleeding after surgery or other hemostatic challenges treated outside the reference center, patients should be instructed to provide feedback to reevaluate the management. The IWG recommends that pedigree investigation with conventional laboratory tests or genetic tests is discussed with the index case. In general, the IWG advises against pervasive genetic testing in clinical practice, apart from targeted exceptions. In some cases, ethical, legal, and social considerations may arise, requiring the assistance of an expert ethicist.

### Bleeders of unknown cause

These patients are at permanent risk of bleeding and pose serious difficulties in counseling and management. Currently, very little is known about their long-term outcomes. The IWG recommends that these patients are treated as if they have a true BD and not dismissed by minimizing their disorder, offering them continued surveillance and assistance. They should be instructed on the use of antifibrinolytics or desmopressin (effective in 90% of cases during minor or major hemostatic challenges^65^) and avoidance of drugs interfering with hemostasis. On the other hand, the IWG agreed that patients with BUC should be reassured since it is unlikely that they have an overlooked severe BD using the proposed multistage approach. The IWG strongly recommends that future clinical, laboratory and genetic research should be devoted to better understand the pathogenetic mechanism and natural course of BUC.

### Women's issues

Bleeding issues in female include heavy menstrual bleeding (HMB), ovulation bleeding, excessive and prolonged bleeding after miscarriage and primary and secondary post-partum hemorrhage (PPH). HMB is a common problem; in a European survey 27% of 4506 women reported HMB, of whom 15% required medical attention.^66^ MBDs (in particular VWD and platelet function disorder) have been shown to be the underlying cause in up to 20% of women with HMB.^67^ PPH, which complicates 5% of deliveries, is often multi-factorial (70% of cases), with uterine atony being the commonest cause. Ovulation bleeding of little clinical significance or occasionally associated with some mid-cycle pain is also common in healthy women - but in women with MBDs such as VWD it may be recurrent or rarely occur as a major bleeding complication. Therefore, testing all such women is not feasible and unnecessary. However, every woman with excessive bleeding should have a risk assessment for underlying BDs. The severity of HMB and of primary or secondary PPH may be difficult to assess quantitatively, particularly when they present after the initiation of hormonal therapy or during pregnancy. In fact, hormones and pregnancy may mask the diagnosis of a bleeding disorder falsely elevating clotting factor or VWF levels. The relatively high values may drop to basal levels after parturition with a risk of bleeding. Decisional thresholds of clotting factors/VWF levels for replacement therapy or reevaluation of diagnosis should be considered. Thus, the value of a thorough past bleeding history and of a family history of bleeding (and historical coagulation factors/VWF levels) cannot be over-emphasized and are the essential tools for presumptive diagnosis. ISTH-BAT criteria and the proposed cut-off of ≥6 (Fig. 1) remain valid for symptoms manifested before the initiation of hormonal therapy prescribed for bleeding. However, the monthly occurrence of annoying bleeding might impact the QoL of some women and fit the definition of nuisance bleeding, thus demanding a full investigation regardless to overall ISTH-BAT score. Similarly, isolated severe gynecological bleeding such as HMB since menarche or acute episodes of HMB or ovulation bleeding requiring hospital admission warrants full investigations for BDs.

HMB and postpartum bleeding are experienced by up to 80% of women with non-severe VWD.^68,69^ Despite replacement therapy at parturition, factor levels drop down rapidly with higher blood loss than in normal women.^70^ The bleeding score predicts clinical outcomes,^70^ but its role in predicting response to replacement therapy, as in non-pregnant individuals,^51^ is not known. It is critical to have a multidisciplinary approach for management with close collaboration with the obstetric/gynecology team and to recognize that the presence of MBD does not rule out the possibility of coexisting gynecological problems. Since first line or single therapy is often not effective for managing HMB in women with BDs, combination therapy including hormonal and antifibrinolytic therapies as well as DDAVP or factor replacement should be considered. In women of reproductive age, a high index of suspicion for iron deficiency (which may impact on QoL) should lead to screening total iron binding capacity (TIBC) and/or ferritin, even when hemoglobin is normal.^71^ For women in families with hemophilia, pedigree analysis and genetic counseling and diagnosis are important for decision-making in relation to reproduction and understanding options for prenatal diagnosis before first pregnancy. The risk of fetal bleeding is minimal with MBD. Management of labor and mode of delivery should be discussed with the mother and planned in advance; precautions should be taken to minimize the risk of fetal trauma during labor and delivery.

## Conclusions

In the absence of general evidence-based outcomes on which to make operative decisions and in the lack of standardized and harmonized definitions, the IWG worked to maximize consensus by proposing clinically relevant definitions, and well-grounded objectives for the investigation of subjects suspected of having a MBD. The strength of the proposal lies in the unanimous panel consensus. This construct has several purposes. First, it proposes a general diagnostic approach to distinguish normal subjects from individuals with a pathologic bleeding tendency, primarily based on a standardized quantitative assessment of the bleeding history. Second, a multistage laboratory approach is proposed for patients with a qualifying phenotype, from standard screening to more specific laboratory or genetic studies. This approach awaits confirmation from the analysis of the subsequent disease-specific evidence-based guidelines. However, the IWG recognizes that some individuals will ultimately not receive a specific diagnosis (BUC), despite a qualifying bleeding phenotype and a full investigation. The IWG proposes this construct to serve as a guide for future development of guidelines specific for distinct MBDs, under the overarching principle of clinical utility.

## Supplementary Material

Supplemental Digital Content
