# Supplementary material for: Fundamentals for a Systematic Approach to Mild and Moderate Inherited Bleeding Disorders: An EHA Consensus Report
Source: Hemasphere. 2019 Sep 17;3(5):e286. doi: 10.1097/HS9.0000000000000286 (PMC6919472; doi:10.1097/HS9.0000000000000286)
Supplement: Supplemental Digital Content [file hs9-3-e286-s001.pdf]

Supplemental Table 1. Provisional working list of known disorders to be considered in the development of future disease-specific guidelines to verify if they may fit the proposed definition of MBD*.

**Disorders**
**Inherited platelet disorders with predominant function impairment**
ADAP (FYB)-related thrombocytopenia
Arthrogryposis, renal dysfunction and cholestasis syndrome
Bernard-Soulier Syndrome (biallelic)
CalDAG-GEFI-related platelet disorder
Chediak-Higashi syndrome
Combined alpha-delta granule deficiency
COX-1 defect
cPLA2 defect
Defect of thromboxane A2 receptor
Defects in GPVI and $\alpha_2\beta_1$
Defects in α2-adrenergic receptor
Delta granule deficiency
Familial platelet disorder and predisposition to acute myelogenous leukemia
FLI-1-related thrombocytopenia
GATA1-related disease
GFI1B-related thrombocytopenia
Glanzmann Thrombasthenia
Gs platelet defect
Tx synthase deficiency
Gray platelet syndrome (platelet count also reduced)
Hermansky-Pudlak syndrome
ITGA2B/ITGB3-related thrombocytopenia
LADIII deficiency
P2Y12 deficiency
Platelet-type von Willebrand Disease
Primary secretion defect
Quebec platelet disorder
Scott syndrome
SLFN14-related platelet disorder
SRC-related defect
Stormorken syndrome
Velocardiofacial syndrome
Tropomyosin 4-related thrombocytopenia
**Inherited platelet number disorders**
ANKRD26-related thrombocytopenia
Bernard-Soulier syndrome (monoallelic)
Congenital amegakaryocytic thrombocytopenia

Congenital thrombocytopenia with radio-ulnar synostosis
CYCS-related thrombocytopenia
ETV6-related thrombocytopenia
FLNA-related thrombocytopenia
MYH9-related disease
Paris-Trousseau thrombocytopenia
Thrombocytopenia with absent radii
TUBB1-related thrombocytopenia
X-linked thrombocytopenia
Wiskott Aldrich Syndrome
**Coagulation disorders**
Von Willebrand Disease types and subtypes, excluding type 3
Mild Hemophilia A
Moderate Hemophilia A
Mild Hemophilia B
Moderate Hemophilia B
Carriers of hemophilia A
Carriers of hemophilia B
**Heterozygous/partial defects of coagulation factors**
Fibrinogen (disorders: hypofibrinogenemia, dysfibrinogenemia)
Factor II or prothrombin (disorders: hypoprothrombinemia, dysprothrombinemia)
Factor V, also called in the past proaccelerin or labile factor or accelerator globulin, terms probably no more used at least since 1975 (disorder: Factor V deficiency, parahemophilia) factor/accelerator globulin
Factor VII, also called serum prothrombin conversion accelerator or stable factor or proconvertin or auto-prothrombin I, terms probably no more used at least since 1975 (disorder: Factor VII deficiency)
Factor X, also called Stuart-Prower factor, term probably no more used at least since 1975 (disorder: Factor X deficiency)
Factor XI, also called plasma thromboplastin antecedent, term probably no more used at least since 1975 (disorder: Factor XI deficiency, hemophilia C)
Factor XIII, also called fibrin stabilizing factor (disorder: Factor XIII deficiency)
**Partial combined coagulation factors deficiencies and complex disorders**
Combined Factors V and VIII
Combined Factors VIII and IX
Combined Factors II, VII, IX and X
Combined Factors VII and VIII
Combined Factors VII and X
Combined Factors VIII, IX and X
Combined Factors IX and XI
Reduced or low or abnormal prothrombin consumption (Scott syndrome, sometimes include in platelet disorders)

East Texas fever
Thrombomodulin gene mutations
**Fibrinolytic disorders**
α2-Plasmin inhibitor deficiency
Plasminogen activator inhibitor-1 (PAI-1) deficiency
**Vascular defects (Ehlers-Danlos syndrome, Osler-Weber-Rendu syndrome, hereditary telangiectasia)**
**Bleeding of unknown cause (BUC)**

* Disorders unanimously considered severe have been excluded upfront. The table is not to be intended as a list of already established MBD. The final retention of these candidate disorders in the group of MBD will be subject to validation on the basis of the evidence gained by the systematic literature review preliminary to the development of the disease-specific guidelines.
